# Recursive regulator: a deep-learning and real-time model adaptation strategy for nonlinear systems

**DOI:** 10.1038/s44172-025-00477-4

**Published:** 2025-08-01

**Authors:** Jinming Sun, Yanqiu Huang, Wanli Yu, Alberto Garcia-Ortiz

**Affiliations:** 1 Institute of Electrodynamics and Microelectronics, University of Bremen, Bremen, Germany; 2 Faculty of Electrical Engineering, Mathematics and Computer Science, University of Twente, Enschede, The Netherlands

**Keywords:** Electrical and electronic engineering, Computational science

## Abstract

Adaptive modeling is imperative for analyzing nonlinear systems deployed in natural dynamic environments. It facilitates filtering, prediction, and automatic control of the target object in real time to respond to unpredictable and non-repetitive sudden physical impairment caused by ambient impacts, such as corrosion, thermal drift, interference, etc. Existing nonlinear modeling approaches, however, are too complex for online training or fall short in rapid model recalibration under such conditions. To address this challenge, here we present a strategy that applies a regulator to the Koopman operator, enabling real-time model adaptation for nonlinear systems. In our approach, the regulator is directly implemented in nonlinear state-space without disrupting the pre-trained black-box predictor. The proposed technique demonstrates efficacy in capturing a broad spectrum of nonlinear dynamics and exhibits rapid adaptability to system changes without requiring offline retraining. Furthermore, its lightweight implementation and high-speed performance make it well-suited for embedded systems and applications demanding fast model recalibration and robustness.

## Abstract (web summary)

Jinming Sun and colleagues propose a real-time model adapting method for nonlinear systems in dynamic scenarios. Its lightweight implementation and high-speed performance make it well-suited for embedded systems and applications demanding fast model recalibration and robustness.

## Introduction

Real-time model adaptation stands as a pivotal foundation for comprehending system behavior under sudden ambient impacts and devising effective prediction and control strategies to maintain system stability and accuracy. For instance, in the field of aerospace systems, automotive systems, structural health monitoring, and communication systems, the intricate nature factors such as exposure to unexpected electromagnetic interference, seismic activity, dynamic loading, and biofouling can cause physical fraying, corrosion, thermal drift, material fatigue, and contamination. These impacts will affect the system dynamics in a sudden and unpredictable way, e.g., increasing the resistance in electronic components, altering the mass of mechanical parts, or changing the damping coefficients. The physical impairments may not be severe enough to result in the need for component replacement or system failures, but they often render the original model inadequate, potentially leading to a loss of control^1^. Facing such challenges, rapid model recalibration becomes apparent as a fundamental requirement for ensuring the reliability of the operational plants. In real-world applications, the inherent complexity of nonlinear dynamics, coupled with the significant costs of model training, poses further challenges for embedded systems that demand real-time responses at low cost^2^. For example, if an autonomous underwater vehicle operating in a nutrient-rich coastal area experiences a rapid and extensive buildup of biofouling due to a sudden algal bloom, this can cause an immediate increase in drag. The vehicle may suddenly struggle to maintain its programmed speed and trajectory, leading to increased energy consumption and potential mission failure. By adapting to the increased drag and altered hydrodynamics caused by biofouling, the system can adjust its propulsion and navigation strategies to maintain operational efficiency. In response to these challenges, various nonlinear modeling and model adaptation methods have been developed.

Prior studies have achieved notable success in constructing nonlinear models for relatively static conditions. Prevailing strategies often simplify the identification process by assuming the system satisfies predefined model structures^3^, or by relying on existing physical knowledge^4^. When addressing the generality for modeling nonlinear systems, deep-learning neural networks (NNs)^5^ emerge as a prominent framework, offering a black-box structure capable of capturing a wide range of nonlinear dynamics. NNs have shown promise in refining simulation error^6^ and facilitating control design^7,8^. While deep learning methods can capture complex dynamics, they demand substantial data and massive computational resources^9,10^. Hence, researchers have increasingly turned to higher-dimensional linear approximation techniques, instead of linearizing the nonlinear dynamics directly. Many works effectively recast the problem in higher-dimensional spaces, where the complexities of nonlinearity can be managed with linear tools, such as lifting transformations^11,12^, Carleman linearization^13^ and Koopman operator theory^14^, which uses a linear operator in an infinite-dimensional space to represent the state evolution of a nonlinear system. However, these techniques generally lack direct adaptability to real-time changes.

In real-world scenarios, sudden impacts often impair the system’s physical components and challenge model robustness. While a range of methods exist for model adaptation, most are designed to handle predictable changes or only pertain to variation in input data^15^, and they operate in an offline set-up. For example, some strategies tackle model variability by offline training for potential scenarios and online selection of candidate^16–18^, but many real-world processes are unpredictable and non-repetitive (e.g., collision, thermal drift). The inability to preemptively obtain varying parameters for training impedes the effectiveness of these approaches. Whilst some deep-learning network structures, such as the recurrent neural networks^19^ and the block-oriented network structures^20,21^, offer online adaptability, their iterative back-propagation procedures and resource-intensive training hinder their suitability for applications that demand cost efficiency and time-critical decisions. Dynamic mode decomposition (DMD)^22^ is a powerful tool when addressing dynamic systems, but it typically works with data batches, or “snapshots”. Recursive DMD (RDMD) is designed for real-time applications through various approximations. However, DMD, including RDMD, inherently assumes a linear system model and can miss finer nonlinear details. Extended dynamic mode decomposition (EDMD), a data-driven approximation of the Koopman operator, is designed to handle nonlinear systems^23^. Like the DMD, EDMD is primarily considered an offline method^24^, as it extracts dynamics from a dataset collected over time, which typically involves processing all the data at once. Recent advancements in real-time updating the Koopman operator have emerged, such as the first-order modification of Koopman operator^25^, incorporating higher-order derivatives in the observables^26^, training deep networks as observable functions^27^, using Fourier filter to disentangle the time-variant components^28^, and Hankel matrix implicit-lifting approximation^29^. These Koopman operator approximation approaches either rely heavily on a rich dictionary of observables that are crucial for ensuring system accuracy and stability, often need to be carefully crafted and usually task-customized; Or they refine the model using “sliding window" data rather than continuously updating with individual data in real-time.

Building upon the successful usage of Koopman operator theory, we propose an efficient black-box recursive structure, the “regulator", for nonlinear model online adaptation when encountering ambient impacts. Our approach eliminates the need for explicit lifting function design, offering a more flexible solution for real-time model adaptation. This paper presents compelling contributions to the field of real-time model recalibration for nonlinear systems, and especially for resource-constrained embedded systems, outlined as follows:

- Recursive regulator to update the Koopman operator: Established in the higher-dimensional linear space of observables, the recursive regulator serves as an amendment to the static-trained Koopman operator predictor, which is embodied by a deep-learning black-box technique. This adapts the nonlinear state space trajectory with impressive accuracy.
- Direct implementation in the finite nonlinear state space: Instead of operating the regulator in the high-dimensional linear space, we offer to reconstruct the regulator in the nonlinear state space. This significantly reduces the dimension for a lightweight implementation, which can be realized by existing linear techniques. This reconstruction makes it possible for the regulator to update independently and rapidly without disruption to the static-trained nonlinear predictor. Therefore, this regulator is particularly suited for applications that demand rapid model responses and low-cost computing.
- Performance evaluation on real-world systems: The efficacy of the proposed recursive regulator is systematically assessed on complex real-world systems. For systems characterized by variability, such as the Electro-mechanical Positioning System (EMPS)^30^, the nonlinear RLC system^31^, and the spring-mass-damper system^3^, the regulator exhibits exceptional accuracy and speed in real-time model adaptation, eliminating the necessity for offline retraining.

## Methods

### Background

This section presents a brief overview of the nonlinear dynamics and the Koopman operator framework, which provides the foundation for our regulator design.

We begin with a representative formula for general nonlinear discrete systems:

1 $${x}_{t+1}=f({x}_{t},{u}_{t})$$

Here, ${x}_{t}\in {{\mathbb{R}}}^{n}$ represents the system state at time *t* in the nonlinear state space. ${u}_{t}\in {{\mathbb{R}}}^{p}$ denotes the system input. $f\in {{\mathbb{R}}}^{n}\times {{\mathbb{R}}}^{p}\to {{\mathbb{R}}}^{n}$ denotes the nonlinear dynamics that moves *x*_*t*_ to the next state *x*_*t*+1_. The system’s parameters *θ* will be introduced later in Eq.(7).

Rather than finding access to the full aspects of the system, we can apply an observable function *h* on the state *x* to obtain system measurable quantities or features of interest (such as position, pressure, etc.) for future prediction and control development. We have the observables *q*,

2 $${q}_{t}=h({x}_{t})=h\left(\,f({x}_{t-1},{u}_{t-1})\right)$$

where $$q\in {{\mathbb{R}}}^{m}$$, $$h\in {{\mathbb{R}}}^{n}\to {{\mathbb{R}}}^{m}$$, and *m* → *∞*.

Unlike an autonomous system where the observables can be advanced by an invariant Koopman operator in the space of observables, for a discrete system under control input, there are several Koopman operators corresponding to each one of the admissible inputs, $\left\{{{{{\bf{u}}}}}_{0},{{{{\bf{u}}}}}_{1},\cdots \,,{{{{\bf{u}}}}}_{j},\cdots \,,{{{{\bf{u}}}}}_{p-1}\right\}$, in a nonlinear manner^32^,

3 $$({{{{\mathcal{K}}}}}_{u}h)(x):= h(\,f(x,u))$$

where ${{{{\mathcal{K}}}}}_{u}$ denotes a family of Koopman operators parameterized by the system input *u*. This means, each one of the Koopman operator ${{{{\mathcal{K}}}}}_{{u}_{t}}$ from the family operates within an invariant function space, where ${{{{\mathcal{K}}}}}_{{u}_{t}}$ is linear, i.e., for observable functions *h*_1_ and *h*_2_, ${{{{\mathcal{K}}}}}_{{u}_{t}}({h}_{1}+{h}_{2})={{{{\mathcal{K}}}}}_{{u}_{t}}{h}_{1}+{{{{\mathcal{K}}}}}_{{u}_{t}}{h}_{2}$.

**Fig. 1:**
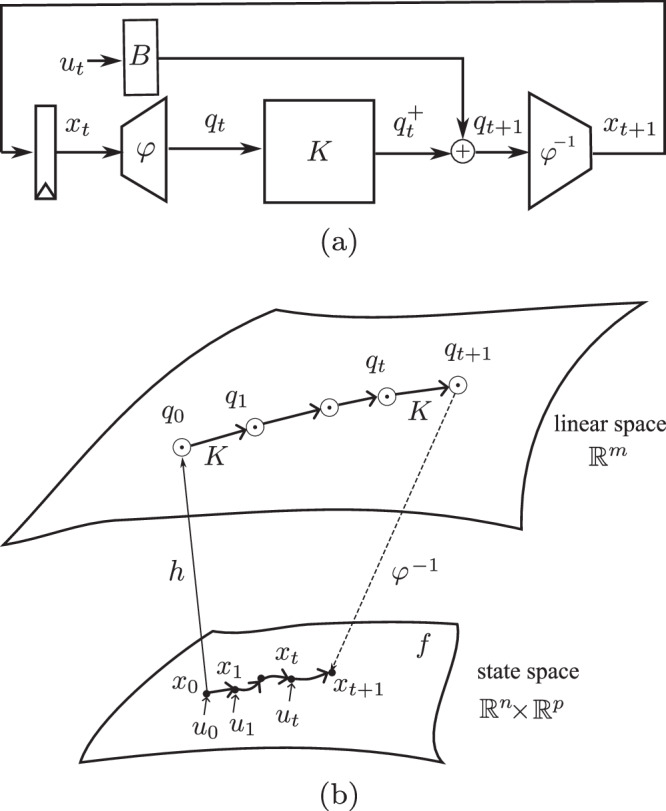
Koopman operator theory with input (finite-dimensional approximation). *f* is a nonlinear dynamics moving the system to the next state in the finite-dimensional nonlinear space. With approximation, the finite-dimensional Koopman matrix *K* forwards observables *q* linearly in the subspace under the influence of input *u*. Using the reverse function *φ*^−1^, we obtain the next state variable *x*_*t*+1_ in the original space.

Since the Koopman operator ${{{{\mathcal{K}}}}}_{u}$ is usually infinite-dimensional, it is necessary in numerical methods that we first consider a finite-dimensional approximation, so that it can be represented by a matrix *K*_*u*_ parameterized by *u*. Then to approximate the lifted state, which still nonlinearly depends on the input^32^, one common approach is selecting a suitable and finite basis of observables, so as to project and further approximate the Koopman operator onto an invariant subspace. In practical and data-driven scenarios for nonlinear discrete systems, it is well accepted to choose both the state-dependent basis ${\left\{{\varphi }_{i}\right\}}_{i = 0}^{m-1}$ and the basis of input ${\left\{{{{{\bf{u}}}}}_{j}\right\}}_{j = 0}^{p-1}$^32^. Thus, in this subspace, as depicted in Fig. 1, the observables are approximated with a linear time-invariant (LTI) dynamics,

4 $${q}_{t+1}\approx K{q}_{t}+B{u}_{t}$$

where observables $q\in {{\mathbb{R}}}^{m}$ is a vector that evolves linearly via the finite-dimensional approximation Koopman matrix $K\in {{\mathbb{R}}}^{m\times m}$. $B\in {{\mathbb{R}}}^{m\times p}$ is the lifting for input *u* to influence the subspace linear dynamics.

In the domain of Koopman theory, the eigenfunctions are frequently discussed. Although the Koopman operator eigenfunctions reflect a particular perspective on the nonlinear system dynamics, their practical utility in modeling and analysis is often limited by the challenges associated with their computation and interpretation. The eigenfunctions can be challenging to compute directly, especially for complex systems with high-dimensional state spaces, and they may not have a straightforward physical interpretation. Whereas observable functions are often selected to capture important physical or dynamic features of the system, offering a more practical and meaningful representation to approximate the dynamics, hence are more often chosen by researchers in practical methods^24,33,34^. The eigenfunctions of the Koopman operator form a complete orthogonal set in the linear observable space, the observable functions can be represented by linear combinations of the eigenfunctions, and span a space of observables which is a projected subspace of the eigenspace^14^.

Assume the basis of observables *φ*_*i*_ contains the identity function *ϕ*, that when applied on a vector returns the vector itself, i.e.,

5a $$\phi ({x}_{t})={x}_{t}$$

5b $$\phi ={\left[{\phi }_{1},\cdots ,{\phi }_{r},\cdots ,{\phi }_{n}\right]}^{T}$$

5c $${\phi }_{r}({x}_{t})={x}_{t}[r]$$

here, *x*_*t*_[*r*] represents the *r*_*th*_ element in *x*_*t*_.

Thus, after the state is transitioned to the linear space, the original state *x*_*t*_ can be retrieved using *ϕ*,

6 $${\varphi }^{-1}({q}_{t}):= \phi ({x}_{t})=C{q}_{t}={x}_{t}$$

This asserts the existence of a linear reconstruction of the original space state *x* from the higher-dimensional space. The reconstruction is defined as *φ*^−1^. Please note that the function *φ* is a coordinate mapping, not a square matrix. Hence the notation *φ*^−1^ is not an inverse of *φ*, but a reverse operation, which is a linear combination of the observables *φ*_*i*_(*x*_*t*_) and manifests in a matrix $C\in {{\mathbb{R}}}^{n\times m}$.

### Recursive regulator: design

The recursive regulator is a rapid model adaptation technique to recalibrate a trained model for the impaired nonlinear system after encountering ambient impacts. This section illustrates the design of the regulator.

Define a vector *θ* that aggregates all the system physical parameters and remains stationary under the original condition. The system dynamic *f*_*θ*_ is parameterized by *θ*,

7 $${x}_{\theta ,t+1}={f}_{\theta }({x}_{\theta ,t},{u}_{t})$$

Our recursive regulator is designed specifically for the models that are based on Koopman framework and employ the LTI approximation in (4). It is rewritten here and parameterized by *θ*,

8a $${q}_{\theta ,t}=h({x}_{\theta ,t})$$

8b $${q}_{\theta ,t}^{+}={K}_{\theta }{q}_{\theta ,t}$$

8c $${q}_{\theta ,t+1}={q}_{\theta ,t}^{+}+{B}_{\theta }{u}_{t}$$

where under the original system condition *θ* = *θ*_0_, simpler notations are used,

9a $${x}_{{\theta }_{0},t}={x}_{t}$$

9b $${q}_{{\theta }_{0},t}={q}_{t}$$

9c $${q}_{{\theta }_{0},t}^{+}={q}_{t}^{+}$$

9d $${K}_{{\theta }_{0}}=K$$

9e $${B}_{{\theta }_{0}}=B$$

When the system is subjected to sudden ambient impacts, its physical components may be slightly impaired, represented as *θ* = *θ*_0_ + Δ*θ*.

a) *Assumption* The observable function *h* is assumed to have formed a sufficiently expressive basis to approximate the Koopman invariant subspace.

b) *Assumption* The impairment Δ*θ* is small, such that it induces only smooth changes in the system dynamics. This assumption excludes drastic alterations, such as bifurcations or chaotic behavior.

**Fig. 2:**
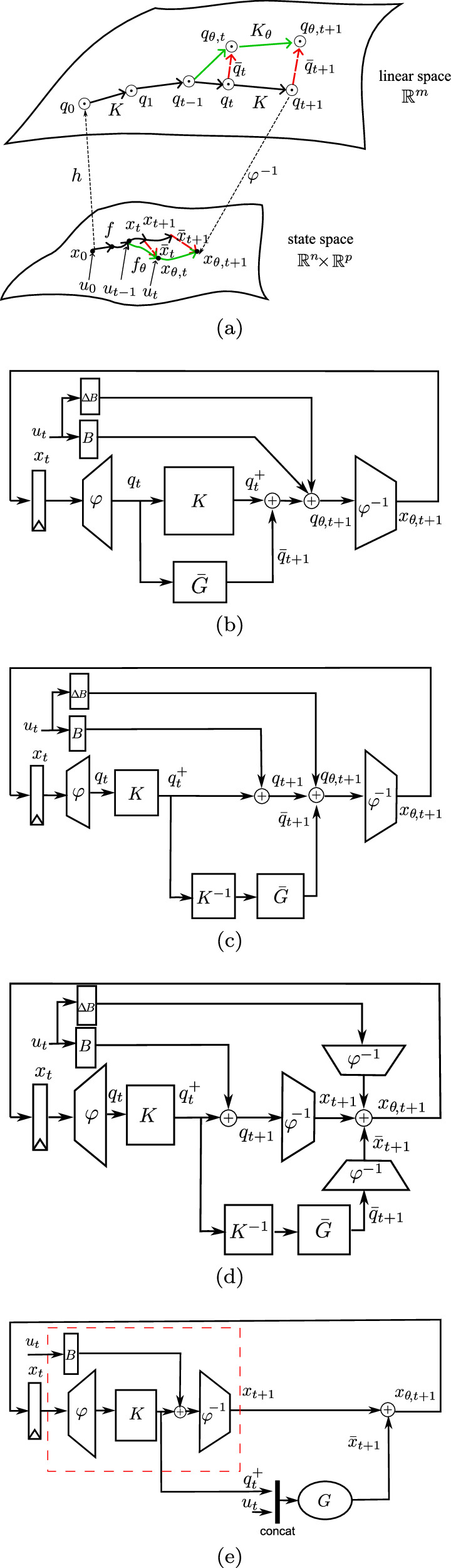
The architecture of the recursive regulator for nonlinear model adaptation. **a** Nonlinear system encountering impacts (green arrows) is observed in a higher-dimensional linear space. **b** The impact influences can be amended via $\bar{G}$ in the observable space. **c** $\bar{G}$ is separated from the Koopman matrix *K*. **d** Decouple $\bar{G}$ from the Koopman predictor structure. **e** A linear regulator *G* can produce the adaptive vector $\bar{x}$ in the original space without any disruption to the Koopman predictor (marked with a red dash box).

As illustrated by the green arrows in Fig. 2a, the original Koopman matrix becomes insufficient when the state ${x}_{{\theta }_{0}}$ deviates to a new state *x*_*θ*_. For this new dynamics, the Koopman operator needs an update. It is important to recognize that the physical impairment may not adhere to a linear process. Whether the ambient impact manifests as a linear or nonlinear influence, we observe and characterize it in the observable space. As with the original system in (4), we approximate the new lifted dynamics using the LTI model. With the assumptions a) and b), the parameter variations are assumed to technically only affect the approximation matrices *K*_*θ*_ and *B*_*θ*_. The updated lifted dynamics can therefore be written as,

10 $$h({x}_{\theta ,t+1})={K}_{\theta }h({x}_{\theta ,t})+{B}_{\theta }{u}_{t}$$

here, *K*_*θ*_ and *B*_*θ*_ are the updated Koopman matrices that operate under the dynamics of the impaired system. They can, of course, be obtained using a data-driven method. However, as the unpredictable ambient phenomenon occurs abruptly, this update needs to be online and rapid, which makes the re-computing less appealing in practice. Therefore, under the assumptions a) and b), we can approximate the update to the Koopman operator, without changing the observable functions or the observable subspace. Approximate *K*_*θ*_ and *B*_*θ*_ around *θ*_0_,

11a $${K}_{\theta }\approx {K}_{{\theta }_{0}}+\frac{\partial K}{\partial \theta }\Delta \theta =K+\bar{G}$$

11b $${B}_{\theta }\approx {B}_{{\theta }_{0}}+\frac{\partial B}{\partial \theta }\Delta \theta =B+\Delta B$$

In the linear observable space, we incorporate a first-order derivative approximation $\bar{G}$ and Δ*B* to adjust the initial Koopman operator to capture the system’s response to the impact-induced dynamics. The increment $\bar{G}$ does not assume a linear response but instead reflects the deviation needed to align *K* with the altered system state, accommodating potentially nonlinear changes due to the impacts.

We now have,

12a $$h({x}_{\theta ,t+1})=(K+\bar{G})h({x}_{\theta ,t})+(B+\Delta B){u}_{t}$$

12b $${q}_{\theta ,t+1}=(K+\bar{G}){q}_{\theta ,t}+(B+\Delta B){u}_{t}$$

#### Remark

The sufficiency of updating the Koopman operator without altering the observable space relies on the premise that the chosen observables form a sufficiently expressive basis to approximate the Koopman-invariant subspace for the system’s dynamics. Smooth parameter variations typically result in modifications to the operator, not to the basis of observables, as long as the new dynamics remain within the span of the original space. This has been validated in studies demonstrating that the Koopman operator adapts efficiently to changes in dynamics when updated with new data, without requiring a redefinition of the observable space^24,33^. Based on our empirical evidence where the observable space is designed with sufficient generality, upon smooth parametric changes that do not involve drastic scenarios (e.g., bifurcations or chaos), the incremental adaptation on the Koopman operator suffices without needing to extend the observable space. Notably, this assumption is implicitly accepted when employing projection-based approximations.

The system changes at time *t*,

13 $${q}_{\theta ,t}={q}_{t}$$

The new observables *q*_*θ*,*t*_ evolve linearly in the observable space via *K*_*θ*_, from (12) and (13),

14a $${q}_{\theta ,t+1}=(K+\bar{G}){q}_{t}+(B+\Delta B){u}_{t}$$

14b $$=K{q}_{t}+B{u}_{t}+\bar{G}{q}_{t}+\Delta B{u}_{t}$$

14c $$={q}_{t+1}+{\bar{q}}_{t+1}+\Delta B{u}_{t}$$

where $${\bar{q}}_{t+1}=\bar{G}{q}_{t}$$.

As depicted in Fig. 2b, this matrix $\bar{G}$ generates an amendment vector ${\bar{q}}_{t+1}$ to update the insufficient state advancement from the initial Koopman matrix. We continue making the following transformations: combining (14) and (8),

15 $${\bar{q}}_{t+1}=\bar{G}{K}^{-1}{q}_{t}^{+}$$

This serves to separate the matrix $\bar{G}$ from the Koopman matrix *K* within the linear space, as depicted in Fig. 2c.

From (6) we obtain the prediction of the system state under original condition, as indicated in Fig. 2d,

16 $${x}_{t+1}={\varphi }^{-1}({q}_{t+1})=C{q}_{t+1}$$

We then reconstruct the calibration $({\bar{q}}_{t+1}+\Delta B{u}_{t})$ back in the nonlinear space by applying *φ*^−1^,

17a $${\varphi }^{-1}({\bar{q}}_{t+1}+\Delta B{u}_{t})={\varphi }^{-1}(\bar{G}{K}^{-1}{q}_{t}^{+}+\Delta B{u}_{t})$$

17b $$=\left[\begin{array}{c}C\bar{G}{K}^{-1}\quad C\Delta B\end{array}\right]\left[\begin{array}{c}{q}_{t}^{+}\\ {u}_{t}\end{array}\right]$$

17c $$=G\left[\begin{array}{c}{q}_{t}^{+}\\ {u}_{t}\end{array}\right]={\bar{x}}_{t+1}$$

where $$G=\left[\begin{array}{c}C\bar{G}{K}^{-1}\quad C\Delta B\end{array}\right]$$ is the regulator to reconstruct an adaptive vector $\bar{x}$ directly in the nonlinear state space. We name this reconstruction the regulator *G*, as depicted in Fig. 2e.

Applying *φ*^−1^ to both sides of (14), leads us to the final nonlinear state *x*_*θ*,*t*+1_:

18 $${x}_{\theta ,t+1}={x}_{t+1}+{\bar{x}}_{t+1}$$

We have now reconstructed the linear regulator, *G*, operating directly in the nonlinear state space. The red box in Fig. 2e outlines the original structure of the Koopman operator prediction framework. Once this predictor is established in a static condition, its structure can remain unchanged and non-disrupted during online operation even in situations of ambient impacts.

Let’s use a simple example^35^ to illustrate the core idea of the regulator,

19a $${x}_{t}=\left[\begin{array}{c}{x}_{1,t}\\ {x}_{2,t}\,\end{array}\right]$$

19b $$\left[\begin{array}{c}{x}_{1,t+1}\\ {x}_{2,t+1}\,\end{array}\right]=\left[\begin{array}{c}\gamma ({x}_{1,t}-{x}_{2,t}^{2})+\beta {u}_{t}\\ \mu {x}_{2,t}\end{array}\right]$$

For this case, the space of observables is finite and spanned by a basis of,

20a $$h({x}_{t})={q}_{t}=\left[\begin{array}{c}{x}_{1,t}\\ {x}_{2,t}\\ {x}_{2,t}^{2}\end{array}\right]$$

20b $$\left[\begin{array}{c}{x}_{1,t+1}\\ {x}_{2,t+1}\\ {x}_{2,t+1}^{2}\end{array}\right]=\left[\begin{array}{ccc}\gamma \quad &0\quad &-\gamma \\ 0\quad &\mu \quad &0\\ 0\quad &0\quad &{\mu }^{2}\end{array}\right]\left[\begin{array}{c}{x}_{1,t}\\ {x}_{2,t}\\ {x}_{2,t}^{2}\end{array}\right]+\left[\begin{array}{c}\beta \\ 0\\ 0\end{array}\right]{u}_{t}$$

where

21 $$K=\left[\begin{array}{ccc}\gamma \quad &0\quad &-\gamma \\ 0\quad &\mu \quad &0\\ 0\quad &0\quad &{\mu }^{2}\end{array}\right],B=\left[\begin{array}{c}\beta \\ 0\\ 0\end{array}\right]$$

And with reverse mapping *φ*^−1^, we reconstruct the original state from the observables,

22 $${x}_{t+1}= 	{\varphi }^{-1}\left({q}_{t+1}\right)=C\,{q}_{t+1}\\ = 	\left[\begin{array}{c}1\quad 0\quad 0\\ 0\quad 1\quad 0\end{array}\right]\left[\begin{array}{c}{x}_{1,t+1}\\ {x}_{2,t+1}\\ {x}_{2,t+1}^{2}\end{array}\right]=\left[\begin{array}{c}{x}_{1,t+1}\\ {x}_{2,t+1}\end{array}\right]$$

The system’s physical components undergo changes due to exposure to ambient impacts, represented by *θ* = [*γ* *μ* *β*]^*T*^ with,

23a $$\gamma ={\gamma }_{0}+\Delta \gamma$$

23b $$\mu ={\mu }_{0}+\Delta \mu$$

23c $$\beta ={\beta }_{0}+\Delta \beta$$

where Δ*γ*, Δ*μ* and Δ*β* represent the variations of the physical parameters after impacts.

Upon incorporating the regulatory strategy, the updated operator in the linear space becomes:

24a $${K}_{\theta }\approx K+\bar{G}=\left[\begin{array}{ccc}{\gamma }_{0}+\Delta \gamma &0&-({\gamma }_{0}+\Delta \gamma )\\ 0&{\mu }_{0}+\Delta \mu &0\\ 0&0&{\mu }_{0}^{2}+2{\mu }_{0}\Delta \mu \end{array}\right]$$

24b $${B}_{\theta }=B+\Delta B=\left[\begin{array}{c}{\beta }_{0}+\Delta \beta \\ 0\\ 0\end{array}\right]$$

where the regulatory mechanism is introduced by leveraging matrices $\bar{G}$ and Δ*B* in Fig. 2b,

25 $$\bar{G}=\left[\begin{array}{ccc}\Delta \gamma \quad &0\quad &-\Delta \gamma \\ 0\quad &\Delta \mu &0\\ 0\quad &0\quad &2{\mu }_{0}\Delta \mu \end{array}\right]$$

and

26 $$\Delta B=\left[\begin{array}{c}\Delta \beta \\ 0\\ 0\end{array}\right]$$

Following the process of decoupling and transitioning back to nonlinear space, we reconstruct the regulator $$G=\left[\begin{array}{c}C\bar{G}{K}^{-1}\quad C\Delta B\end{array}\right]$$ as:

27 $$G=\left[\begin{array}{ccc|c}\frac{\Delta \gamma }{{\gamma }_{0}}&0&0 &\Delta \beta \\ 0&\frac{\Delta \mu }{{\mu }_{0}}&0 &0\end{array}\right]$$

The model adaptation matrix *G* can, in principle, be computed through a straightforward least-squares optimization. However, to ensure accurate prediction, it is also necessary to discover a suitable initial condition ${x}_{\theta ,{t}_{0}}$ for the updated model, i.e., to align the trajectory of ${x}_{{\theta }_{0},t}$ with *x*_*θ*,*t*_. In practice, real-world systems generally do not provide access to full state trajectories, offering only the system input *u* and measurement *y*. Given the higher-dimensional data such as *q*^+^, this problem falls within the scope of what a state-space model (SSM) based Prediction Error Method (PEM)^10^ is well suited to address. Because SSM handles multi-input systems naturally and can capture both the input-output relationship and the internal dynamics, i.e., holding a memory, PEM can be used to effectively align the trajectories. Therefore, as proposed in the next section, we use a state space model to achieve the functionality of the regulator *G* and to produce the vector $\bar{x}$ based solely on the available system measurements.

### Recursive regulator: implementation

The previous section outlines the underlying mathematical principles and the architecture of the recursive regulator, this section will translate the design into a tangible, executable framework. The practical implementation of the regulator necessitates an integration of two crucial components, as portrayed in Fig. 2e, a nonlinear predictor (inside the red box) and the regulator *G*.

**Fig. 3:**
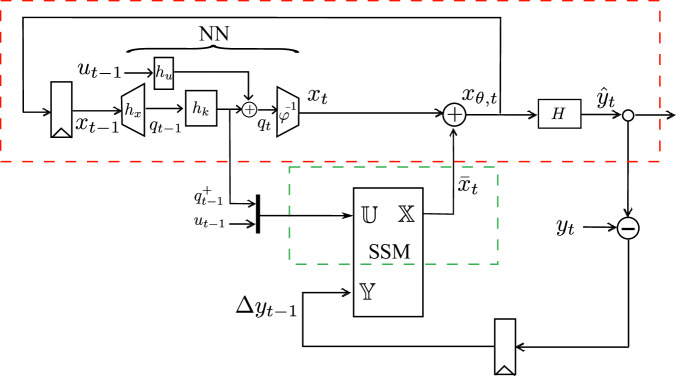
The scheme of the recursive regulator for nonlinear model online adaptation. It consists of a nonlinear predictor (inside the red box) and an SSM regulator (inside the green box). The NN high-dimensional state ${[{q}^{+},u]}^{T}$ goes to SSM. Using the deviation Δ*y* as a reference correction value, the SSM internal variable ${\mathbb{X}}$ serves as an incremental adaptation that amends the inaccuracy in NN's prediction.

As we know, the Koopman operator provides an exact, infinite-dimensional framework for analyzing nonlinear dynamics, but the exact Koopman matrix is often impractical to compute. Therefore, researchers usually employ finite-dimensional, data-driven approximations. In our cases, we use the neural network (NN) structure to approximate the Koopman prediction framework (inside the red box in Fig. 3).

NN creates its own basis functions via learned weights. In this work, the NN structure consists of four blocks: one nonlinear input block for lifting the state *x*_*t*_ to observables *q*_*t*_, a linear layer to advance the observables *q*_*t*_, another input linear layer for lifting the input *u*_*t*_, and a linear output layer to reconstruct the advanced state *x*_*t*+1_.

The state *x*_*t*_ is lifted into a higher dimension by the nonlinear block *h*_*x*_ with weights *W*_*x*_, activation *σ* and bias *b*_*x*_ to obtain observables *q*_*t*_. In our experiments, we have implemented a simple nonlinear layer,

28 $${q}_{t}={h}_{x}({x}_{t})=\sigma ({W}_{x}{x}_{t}+{b}_{x})$$

The nonlinear block can have *l* nonlinear layers, then,

29 $${q}_{t}={h}_{x}^{(i)}({x}_{t})=\sigma ({W}_{x}^{\,(i)}{h}_{x}^{(i-1)}({x}_{t})+{b}^{(i)}),\quad i=2,...,l$$

A linear layer *h*_*k*_ with weights *W*_*k*_ advances the observables to ${q}_{t}^{+}$,

30 $${q}_{t}^{+}={h}_{k}({q}_{t})={W}_{k}{q}_{t}$$

Koopman matrix is implicitly incorporated in the weighted connections that perform data linear advancement.

The input *u*_*t*_ is lifted via a linear layer *h*_*u*_ with weights *W*_*u*_,

31 $${h}_{u}({u}_{t})={W}_{u}{u}_{t}$$

Advanced observables *q*_*t*+1_ are obtained via,

32 $${q}_{t+1}={q}_{t}^{+}+{h}_{u}({u}_{t})$$

The NN output layer *φ*^−1^ with weights *W*_*c*_ performing a linear combination of the hidden layer neurons produces the next system state *x*_*t*+1_,

33 $${x}_{t+1}={\varphi }^{-1}({q}_{t+1})={W}_{c}({q}_{t}^{+}+{h}_{u}({u}_{t}))$$

When using NN to lift the system to observable space, we do not need to manually specify the observables^36^, which can be challenging to select for complex systems.

NN is offline-trained in the static condition, by solving,

34 $${\min }_{W,b}\sum\limits_{t}\parallel {x}_{t}-{\varphi }^{-1}({q}_{t}){\parallel }^{2}$$

to ensure that it fully captures the nonlinearity of the system dynamics.

However, it’s worth noting that this NN configuration represents just one realization of the Koopman operator implementation. Users have the flexibility to select their preferred Koopman approximation predictor based on their specific application requirements.

Once we obtain a nonlinear model under the static condition, i.e., *θ* = *θ*_0_, we can incorporate the regulator *G* (inside the green box in Fig. 3). It is achieved by using a lightweight state space model (SSM), represented as,

35 $${{\mathbb{X}}}_{t+1}={\mathbb{A}}{{\mathbb{X}}}_{t}+{\mathbb{B}}{{\mathbb{U}}}_{t}$$

where ${\mathbb{X}}\in {{\mathbb{R}}}^{{n}_{x}}$, ${\mathbb{U}}\in {{\mathbb{R}}}^{{n}_{q}+{n}_{u}}$ are the state variable and input of SSM, respectively; ${\mathbb{A}}\in {{\mathbb{R}}}^{{n}_{x}\times {n}_{x}}$ and ${\mathbb{B}}\in {{\mathbb{R}}}^{{n}_{x}\times ({n}_{q}+{n}_{u})}$ are the state matrix and input matrix of SSM, respectively. Please note that the input ${\mathbb{U}}$ and state ${\mathbb{X}}$ of SSM are not the general sense of system input *u* and state *x*.

The elements of ${\mathbb{A}}$ and ${\mathbb{B}}$ in SSM are optimized by PEM to minimize model prediction error,

36 $${\min }_{{\mathbb{A}},{\mathbb{B}}}\sum\limits_{t}\parallel {y}_{t}-{\hat{y}}_{t}{\parallel }^{2}$$

where *y*_*t*_ is the system measurement at time *t* and ${\hat{y}}_{t}$ is the prediction of the regulator-embedded nonlinear predictor at time *t*.

Remarkably, by implementing the regulator with PEM, *G* adapts the prediction trajectory *x*_*θ*,*t*_ as well as the Koopman operator for the new system dynamics.

Our model is validated using the coefficient of determination *R*^2^,

37 $${R}^{2}=1-\frac{{\sum }_{t}{(\,{y}_{t}-{\hat{y}}_{t})}^{2}}{{\sum }_{t}{(\,{y}_{t}-{y}^{mean})}^{2}}$$

where *y* ^*mean*^ is the mean of all measurements.

#### Algorithm 1

The recursive regulator online implementation and evaluation.

1: Initialize: *x*_0_

2: Input: *u*_0_ , *u*_1_, . . . , *u*_*t*_

3: Output: $${\hat{y}}_{0},{\hat{y}}_{1},...,{\hat{y}}_{t}$$

4: **while** system online **do**

5: $$\bigtriangleup {y}_{t-1}={y}_{t-1}-{\hat{y}}_{t-1}$$

6: $${x}_{t},{q}_{t-1}^{+}=N\,N({x}_{t-1},{u}_{t-1})$$

7: $${\mathbb{A,\; B}}=P\,E\,M\,(\bigtriangleup {y}_{t-1})$$

8: $${\bar{x}}_{t}={\mathbb{A}}{\bar{x}}_{t-1}+{\mathbb{B}}{[{q}_{t-1}^{+}\,{u}_{t-1}]}^{T}$$

9: $${x}_{\theta ,t}={x}_{t}+{\bar{x}}_{t}$$

10:  $${\hat{y}}_{t}=H{x}_{\theta ,t}$$

11: **end while**

12: Validate: $${R}^{2}(\{{y}_{0},{y}_{1},...,{y}_{t}\},\{{\hat{y}}_{0},{\hat{y}}_{1},...,{\hat{y}}_{t}\})$$

The operational scheme is detailed in Fig. 3 and Algorithm 1. The SSM component operates in conjunction with the pre-trained NN. Within this architecture, each state incrementally builds upon the preceding one. The NN high-dimensional state and system control variable ${[{q}_{t-1}^{+}\,{u}_{t-1}]}^{T}$ goes to SSM as an input ${\mathbb{U}}$ to produce ${{\mathbb{X}}}_{t+1}$, which then serves as an incremental adaptation ${\bar{x}}_{t}$ that amends NN’s inaccurate prediction *x*_*t*_ to achieve an updated state *x*_*θ*,*t*_. With an output matrix *H*, the predictor produces a ${\hat{y}}_{t}$ that is subsequently compared against the measurement *y*_*t*_. The resultant error Δ*y* contributes to the updating of SSM parameters ${\mathbb{A}}$ and ${\mathbb{B}}$.

When using a simple approximation in (4) for the mapping *u* → *K*_*u*_, we inevitably introduce inaccuracy to the model. The scheme in Fig. 3 also helps to account for potential inaccuracies in the modeling of *K*_*u*_. The re-lifting structure (*x*_*θ*,*t*_ fed back into NN) can profoundly influence the trajectory accuracy and internal consistency^37^.

In the online operational scenario, the pre-trained NN proficiently produces predictions given an initial state *x*_0_ and the excitation $\left\{{u}_{0},{u}_{1},\cdots \,,{u}_{t-1},{u}_{t}\right\}$. This process persists in accuracy until the system undergoes sudden ambient impacts and exhibits deviations that render the NN prediction *x* unreliable. Instead of retraining NN, the training of the recursive SSM can effectuate the necessary adaptations denoted by $\bar{x}$ to rectify the veering predictions.

Continuing the example (27), as the state-space representation can yield multiple valid solutions, a simple solution to produce the adaptation ${\bar{x}}_{t+1}$ can be achieved by assuming ${\mathbb{A}}$ is singular, meaning it does not contribute to the dynamics, and ${\mathbb{B}}=G$, therefore,

38a $${\bar{x}}_{t+1}=G\left[\begin{array}{c}{q}_{t}^{+}\\ {u}_{t}\end{array}\right]$$

38b $$=\left[\begin{array}{ccc|c}\frac{\Delta \gamma }{{\gamma }_{0}}&0&0&\Delta \beta \\ 0&\frac{\Delta \mu }{{\mu }_{0}}&0&0\end{array}\right]\left[\begin{array}{c}{\gamma }_{0}({x}_{1,t}-{x}_{2,t}^{\,{2}})\\ {\mu }_{0}{x}_{2,t}\\ {\mu }_{0}^{2}{x}_{2,t}^{2}\\ {u}_{t}\end{array}\right]$$

38c $$=\left[\begin{array}{c}\Delta \gamma ({x}_{1,t}-{x}_{2,t}^{2})+\Delta \beta {u}_{t}\\ \Delta \mu {x}_{2,t}\end{array}\right]$$

For a detailed validation using this example, please refer to the supplementary note.

The strength of our approach lies in the updating independence of the regulator for nonlinear model adaptation. We emphasize a strategy wherein the regulation of the Koopman operator in an observable space can improve the resolution of nonlinear challenges. With our framework, the regulator can be implemented practically in the original space without disrupting the nonlinear predictor.

## Results and discussion

This section assesses the online efficiency of the recursive regulator in terms of prediction accuracy (*R*^2^) and adaptation speed, using real-world nonlinear examples. Specifically, we examine the Electro-mechanical Positioning System (EMPS)^30^, the nonlinear RLC system^31^, and the mass-spring-damper system^3^. These systems not only represent diverse applications within electronics and engineering fields but also model fundamental dynamics across various disciplines. By showcasing the regulator’s versatility and robustness on different dynamic behaviors and impairing effects, we demonstrate its broad applicability. The evaluation of the adaptation speed further highlights the method’s suitability for tasks that demand real-time and cost-effective responses.

### Settings

All processes are conducted on a PC featuring an Intel Core i7-10700 CPU operating at 2.90 GHz and 32.0 GB RAM. In our experiments, the NN predictor consists of four parts: a *ReLU*^38^ activated nonlinear layer with two inputs and 64 neurons to lift the system state *x*, then advanced by a linear layer with 64 neurons; a linear layer with one input and 64 neurons to lift the system excitation *u*; and another linear layer with 64 inputs and 2 outputs to reconstruct in the state space. The output matrix *H* in Fig. 3 uses an identical matrix. The weights are randomly initialized with values from a normal distribution parameterized by *mean* = 0 and *std* = 10^−3^. We employ the forward-Euler procedure for unrolling data in time and use the mean squared error as the loss function. The regulator is characterized by SSM in the form of a second-order “canonical observer”. The recursive PEM^10^ is used for real-time SSM updating in varying scenarios.

Regardless of which nonlinear predictor the user employs, it must be pre-trained offline in a non-varying condition to capture the system’s nonlinearity and optimize the initial state *x*_0_. Importantly, the cost associated with NN offline pre-training is not considered in the online simulation. Since the online simulation begins under the original system condition, the offline-trained initial state *x*_0_ is retained. The discussion of nonlinear predictor hyperparameter optimization^10,31^ is not included in this paper.

### Electro-mechanical positioning system (EMPS)

**Fig. 4:**
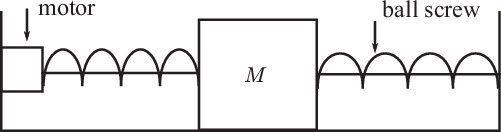
The device of Electro-mechanical positioning system (EMPS)^30^.

The EMPS^30^, depicted in Fig. 4, plays a critical role in applications requiring precise control of position, such as in robotics, industrial automation, precision manufacturing, and medical prosthetic devices. It employs a bang-bang acceleration for mass movement within the device frame, facilitating the identification of parameters like inertia, gravity, and friction. Ambient impacts such as temperature fluctuations, mechanical vibrations, electromagnetic interference, and humidity can cause expansion or contraction of mechanical components, changes in friction, or altered mechanical properties and affect the performance and stability of EMPS.

The underlying dynamics of EMPS are governed by Newton’s law,

39 $$\tau =M\ddot{p}+{F}_{v}\dot{p}+{F}_{c}sign(\dot{p})+f$$

where *p*, $\dot{p}$ and $\ddot{p}$ represent the mass’s position, velocity and acceleration, respectively; *τ* denotes the motor force; *M* is the joint mass, and *F*_*v*_, *F*_*c*_ stand for viscous and Coulomb frictions, respectively; *f* is an offset of *τ*.

For training NN in an offline non-varying environment, the model input *u* and output *y* are motor force *τ* and mass position *p*, respectively. Learning rate is set to 0.0001 in the Adam algorithm when offline training NN through 10,000 epochs. The NN is endowed with three inputs, including two presumed state variables *x* representing *p*, $\dot{p}$ and system excitation *u*, and with one output representing the prediction $\hat{y}$ for position. The implementation parameters in (39) are set as follows: *f* = − 3.1648, *M* = 95.1089, *F*_*v*_ = 203.5034, and *F*_*c*_ = 20.3935. The sampling frequency is *dt* = 0.005 s, the total sample size for training is 3000 and the training time takes up 86.13 s. For testing the regulator, the sampling frequency remains the same (*dt* = 0.005 s), with a total sample size of 14,000. The data *u* and *y* in both datasets are normalized in range [0, 1].

The testing dataset for EMPS is structured into three distinct segments, as outlined in Table 1. The first segment [0, *t*_1_) retains the system’s original physical parameters, but with Gaussian noise introduced into both the process and measurement components. The normal Gaussian distribution has mean 0 and variance 10^−3^. In the second segment [*t*_1_, *t*_2_), the system’s physical components undergo an ambient impact, which degrades key parameters such as mass and damping coefficients. This modification simulates the natural effects that occur in real-world systems. By introducing controlled changes in the parameters, we can validate the robustness and effectiveness of the regulator in real-time model adaptation, ensuring it can handle the dynamic nature of systems. The third segment [*t*_2_, *t*) maintains a constant system physical configuration. However, the reference signal experiences a transition from a sinusoidal waveform to a triangular waveform. This tests the system’s response to altered reference signals, ensuring the regulator’s robustness to both internal changes (due to physical impairment) and external changes (different input types). Such transitions are critical for validating the generalization capabilities and practical applicability of the regulator in real-world scenarios where the system encounters various signal patterns and types. Similar to the previous segments, Gaussian noise is put into the system.

**Table 1:** EMPS varying parameters for testing the recursive regulator. *t*_1_ = 20 s, *t*_2_ = 50 s, *t* = 70 s, *aging*_*factor* = 0.80

Data	System parameters
[0, *t*_1_)	original parameters, Gaussian noise in process and measurement
[*t*_1_, *t*_2_)	*f* = 0.99*f*, *M* = *aging*_*factor* *M, *F*_*v*_ = *aging*_*factor* *F_*v*_, *F*_*c*_ = *aging*_*factor* *F_*c*_, Gaussian noise
[*t*_2_, *t*)	reference signal changes from sin-wave to triangle-wave, Gaussian noise

**Fig. 5:**
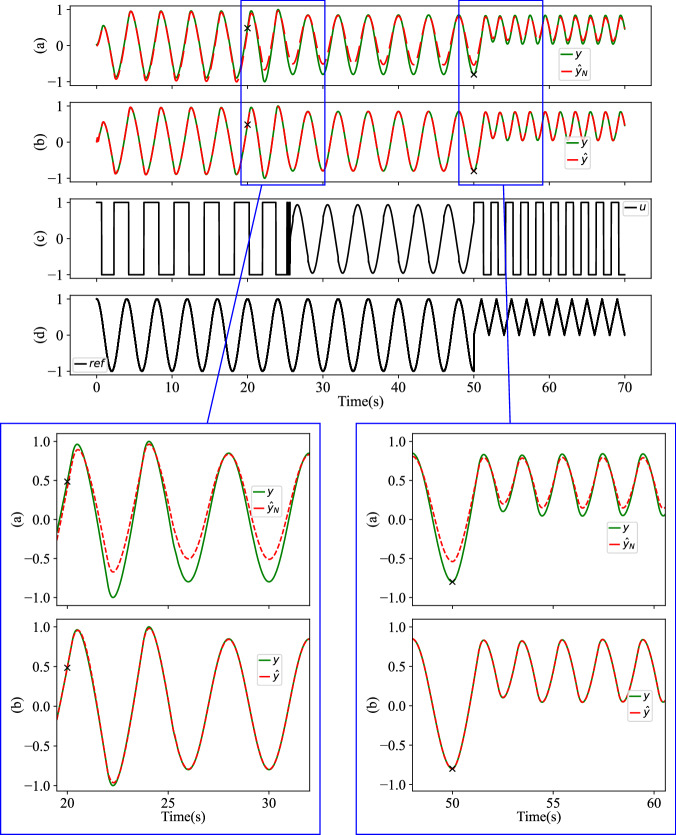
EMPS test result in varying scenarios from Table 1. Changes (marked with  × ) occur in physical parameters and the reference signal. *y*, ${\hat{y}}_{N}$, and $\hat{y}$ represent the actual measurements, the pure pre-trained NN prediction and the regulated prediction, respectively. From top to bottom: **a** Using only the pre-trained NN model, *R*^2^ = 0.955. **b** Applying the recursive regulator, *R*^2^ = 0.999. **c** The system input changes to maintain stability after the system components are impaired. **d** Reference signal changes at time 50 s.

The simulation results based on Table 1 are shown in Fig. 5. The first “×” marks the instance where the system parameters change, and the second “×” denotes the moment of transition in the reference signal.

From Fig. 5a, it is clear that the original NN predictor has limitations when dealing with an impaired system. Moreover, when the reference signal shifts from a sinusoidal waveform to a triangle waveform, despite the system’s physical structure remaining unchanged, the prediction deviates further. In this instance, the overall accuracy is *R*^2^ = 0.955, and the run time amounts to 1.64 s. Figure 5b showcases the performance of the recursive regulator in the same impact situation. The regulated predictions $\hat{y}$ achieve a better accuracy *R*^2^ = 0.999. The run time for the 14,000 samples amounts to 16.09 s, resulting in an execution time of 1.15 ms per sample. This processing speed is well within the system’s sampling interval of *dt* = 5 ms, demonstrating that the regulator is indeed feasible for real-time implementation.

**Fig. 6:**
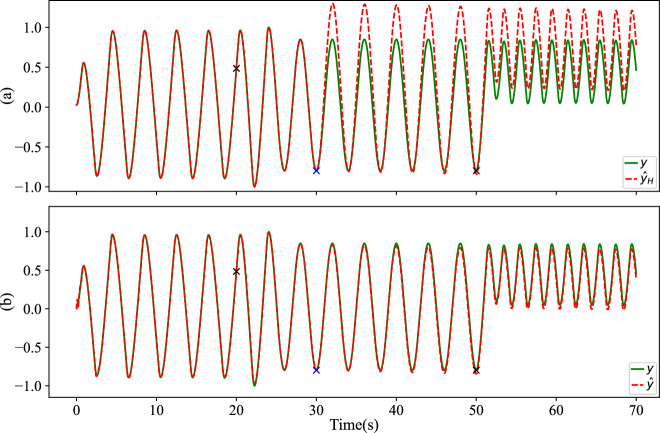
Results comparison between Hammerstein and the regulator for EMPS varying scenarios. The NN component is fixed during online operation. True measurement *y* stops being provided to all models at time 30 s (mark with a blue  × ). From top to bottom: **a** The Hammerstein prediction ${\hat{y}}_{H}$ worsens when online updating stops at 30 s. The overall *R*^2^ = 0.863. **b** The recursive regulator can continue to adjust the nonlinear trajectory $\hat{y}$ until the system changes again at 50 s. The overall *R*^2^ = 0.996.

An existing structure, the block-oriented Hammerstein^39^, which consists of a static nonlinear block *F* followed by a linear time-invariant block *G*, can also model nonlinear dynamic systems. For instance, *F* can be represented by the same neural network, and *G* could be a second-order ARX model^40^. The crucial distinction in our proposed regulator compared to the Hammerstein model or any other filters is that our regulator is a model adaptation rather than signal filtering. With this SSM regulator model adaptation and the re-lifting framework, our structure has a long-range sequential prediction. As compared in Fig. 6 with data from Table 1, at time 30 s (marked with a blue  × ), we stop providing the measurement *y* to all models. From 30 s to 50 s, with the same static-trained NN, the regulator can continue predicting the nonlinear trajectory even when the SSM updating stops at 30 s, until the model needs further adaptation due to the system changing again at 50 s. In contrast, the filter structure, Hammerstein model, ceases to effectively filter the NN output without the true value *y*. However, when the system changes again at time 50 s, without model updating, all methods fail to capture the new dynamics.

Table 2 presents a comparison regarding model accuracy and speed between the recursive regulator and two other state-of-the-art model adaptation methods. The dynoNet^21^ utilizes a neural network structure with linear layers that can be updated independently to adapt to system changes. In this comparison, the retraining iterations for dynoNet are set to 10,000. Although dynoNet achieves a fair accuracy of *R*^2^ = 0.970, it has the longest runtime, as the updating process relies on back-propagation, which requires a significant number of iterations. This extended runtime limits its suitability for online applications that require rapid response and may be impractical for embedded systems due to resource constraints. The EDMDc^36^, in this case, employs radial basis functions as observables and updates the model using a batch data size of 4000 in a sliding-window fashion. While EDMDc achieves excellent accuracy comparable to the recursive regulator, its batch-based updating approach results in a longer runtime than the recursive regulator. Notably, the runtime efficiency of our recursive regulator can be more evident if implemented on embedded systems, where the memory and computational constraints become limiting factors, instead of on a PC.

**Table 2:** Model adaption methods comparison

Methods	*R*^2^	Runtime (14,000 samples)
dynoNet^21^	0.970	104.43 s (10,000 iterations)
EDMDc^36^	0.999	33.40 s
Recursive Regulator	0.999	16.09 s

These results underline the recursive regulator’s capacity to adapt the imprecise nonlinear model caused by system physical impairments. They highlight its effectiveness in adaptation while keeping computational overhead within acceptable limits to provide rapid response.

This regulator framework has generic applicability. We demonstrate its versatility using two additional examples, which also have significant relevance in engineering and other scientific fields.

### Nonlinear RLC

**Fig. 7:**
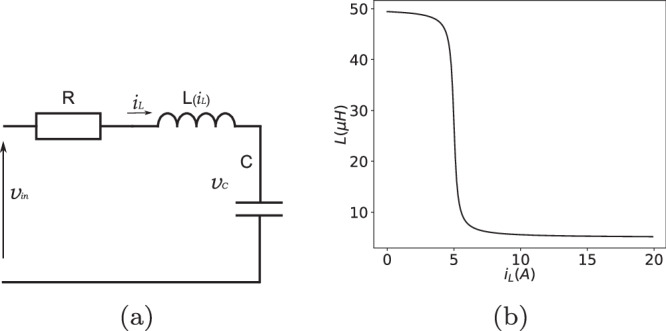
The nonlinear RLC system. **a** Nonlinear RLC model. **b** The inductance *L* depends nonlinearly on *i*_*L*_.

The nonlinear RLC circuit^31^ in Fig. 7 represents a basic electronic model and is used in countless applications. This system is chosen as an example because the ambient impact can significantly affect its electrical properties. Resistance may change due to corrosion, material degradation, and sudden temperature changes. Capacitance can change as a result of dielectric degradation, physical changes, and temperature effects. Inductance may alter due to core material changes, physical wear, and sudden external magnetic fields. Understanding and being able to model these effects are crucial for the control and maintenance of electrical and electronic systems, ensuring their reliability and longevity.

The system is described as,

40a $$\left[\begin{array}{c}\dot{{v}_{C}}\\ \dot{{i}_{L}}\end{array}\right]=\left[\begin{array}{c}0\qquad \frac{1}{C}\\ \frac{-1}{L({i}_{L})}\quad \frac{-R}{L({i}_{L})}\end{array}\right]\left[\begin{array}{c}{v}_{C}\\ {i}_{L}\end{array}\right]+\left[\begin{array}{c}0\\ \frac{1}{L({i}_{L})}\end{array}\right]{v}_{in}$$

40b $$L({i}_{L})={L}_{0}[0.9(\frac{1}{\pi }\arctan (-5(| {i}_{L}| -5)+0.5)+0.1)]$$

where system excitation *v*_*in*_(V) is a filtered white noise. *v*_*C*_(V) is the capacitor voltage. *i*_*L*_(A) is the inductor current. And inductance *L* depends nonlinearly on *i*_*L*_.

For offline training NN, the signals *v*_*in*_ and *v*_*C*_ are taken as model input *u* and output *y*, respectively. Learning rate is set to 0.001 in the Adam algorithm when NN is trained through 10,000 epochs taking up 191.08 s. The NN is endowed with three inputs, including two state variables *x* representing *v*_*C*_, *i*_*L*_ and system excitation *v*_*in*_, and with one output representing the prediction $\hat{y}$ for *v*_*C*_. The initial implementation parameters are set as follows: the input white noise *v*_*in*_ has bandwidth *bw* = 30 kHz and standard deviation *std* = 80 V; *R* = 3 Ω, *C* = 270 nF, and *L*_0_ = 50 μH^41^. The training data set is generated for 2 ms with sampling frequency *dt* = 0.5 μs.

To evaluate the efficiency of the proposed recursive regulator, we collect the test data for 3 ms with the same *dt* = 0.5 μs. The circuit parameters are modified into four distinct segments, as listed in Table 3, to strategically emulate the ambient impact that the system can encounter during online operation. The additional normal Gaussian distribution has mean 0 and variance 10^−3^. The input-and-output data *u* and *y* are normalized into [0, 1].

**Table 3:** RLC varying parameters for testing the recursive regulator

Data	System parameters
[0, *t*_1_)	original parameters, Gaussian noise in process and measurement
[*t*_1_, *t*_2_)	*bw* = 35 kHz, *std* = 60 V, *R* = 7 Ω, *C* = 170 nF, *L*_0_ = 40 μH, Gaussian noise
[*t*_2_, *t*_3_)	*bw* = 10 kHz, *std* = 70 V, *R* = 14 Ω, *C* = 100 nF, *L*_0_ = 30 μH, Gaussian noise
[*t*_3_, *t*)	*bw* = 20 kHz, *std* = 30 V, *R* = 17 Ω, *C* = 70 nF, *L*_0_ = 20 μH, Gaussian noise

*t*_1_ = 0.5 ms, *t*_2_ = 1 ms, *t*_3_ = 2.5 ms, *t* = 3 ms.

The first segment [0, *t*_1_) retains the original circuit’s physical parameters. To simulate real-world scenarios, Gaussian noise is intentionally introduced into both the process and measurement components of the system. In the next three segments [*t*_1_, *t*), the circuit’s physical components undergo several ambient impacts. Key parameters such as resistance, capacitance, and inductance are modified to replicate the impairments. Additionally, Gaussian noise is applied to mimic variations more realistically. The excitation signal has varying bandwidth and standard deviation, thereby challenging the system’s response to altered input signals.

**Fig. 8:**
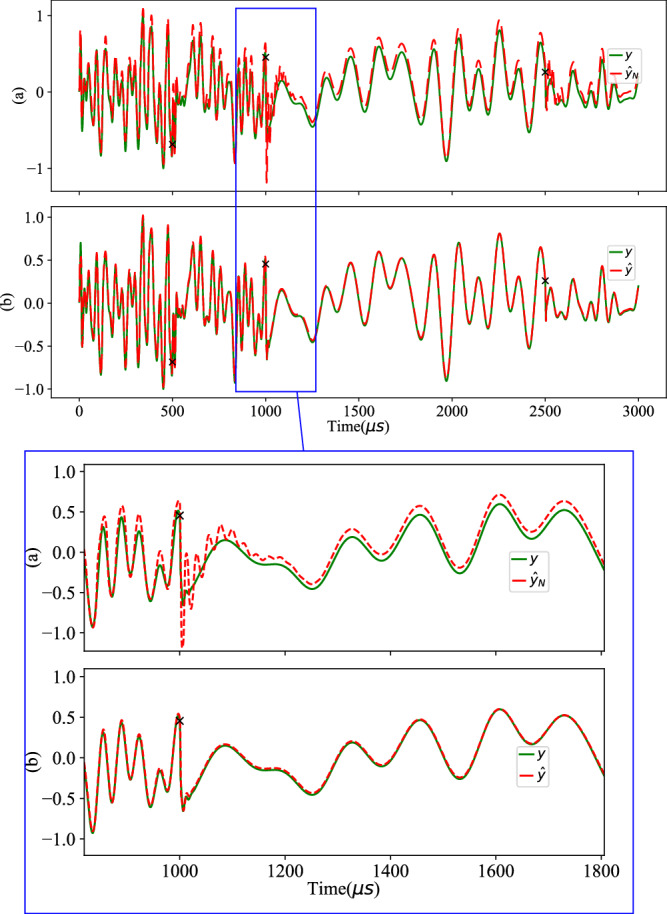
Nonlinear RLC test result for impact scenarios from Table 3. Impairments occur in physical parameters (marked with  × ). *y*, ${\hat{y}}_{N}$, and $\hat{y}$ represent the actual measurements, the pure pre-trained NN prediction and the regulated prediction, respectively. From top to bottom: **a** Using only the pre-trained NN model, *R*^2^ = 0.904. **b** Applying our recursive regulator, *R*^2^ = 0.994. Data *u* and *y* are normalized in range [0, 1].

The simulation results based on Table 3 are visualized in Fig. 8. The “ × ” marks the instances where the system parameters undergo impacts. The green line represents the actual measurements *y*, whereas the red dashed line represents the pure pre-trained NN prediction ${\hat{y}}_{N}$ and the regulator prediction $\hat{y}$. The test result makes it evident that the original model’s predictions exhibit discrepancies from the real measurements as soon as the system parameters change at *t*_1_. As shown in Fig. 8a, the NN predictor’s performance worsens as the system changes again at *t*_2_ and *t*_3_. This divergence highlights the limitation of the static-trained model, which no longer accurately captures the system’s behavior after the impacts. In this scenario, the overall accuracy is *R*^2^ = 0.904, and the runtime amounts to 0.55 s. Conversely, in Fig. 8b, the recursive regulator intervenes effectively. The regulated prediction $\hat{y}$ converges fast towards a better accuracy, achieving an impressive *R*^2^ = 0.994. Despite the incorporation of the SSM component, the runtime experiences only a slight increase to 6.91 s, which suffices for a reasonably rapid adaptation to maintain operational stability.

### Mass-spring-damper system

**Fig. 9:**
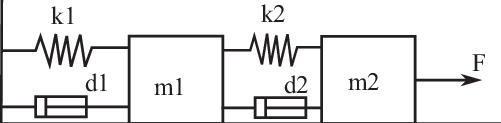
The mass-spring-damper system. Two objects *m*_*1*_ and *m*_*2*_ are connected by springs and dampers. The springs have constants *k*_*1*_ and *k*_*2*_, and the dampers have constants *d*_*1*_ and *d*_*2*_. An exterior force *F* is acting on the object *m*_*1*_.

The mass-spring-damper system^3^ effectively models dynamic behaviors, oscillations, and responses to forces, making it valuable in engineering, physics, and related fields. For example, it is used for analyzing human motion, and the vibration of mechanical structures (bridges, buildings, vehicles, etc) and equipment (machinery, sports equipment, etc). As depicted in Fig. 9, it consists of two masses connected by springs and dampers.

Newton’s second law governs the dynamics,

41a $${m}_{1}{\ddot{x}}_{1}={f}_{k2}+{f}_{d2}-{f}_{k1}-{f}_{d1}-{F}_{c}sign(\dot{{x}}_{1})$$

41b $${m}_{2}{\ddot{x}}_{2}=F-{f}_{k2}-{f}_{d2}-{F}_{c}sign(\dot{{x}}_{2})$$

where *x*_1_, *x*_2_ denote the displacements of two objects with masses *m*_1_, *m*_2_, respectively; *f*_*k*1_, *f*_*k*2_ represent the forces of springs with spring constants *k*_1_, *k*_2_, respectively; *f*_*d*1_, *f*_*d*2_ are the forces of dampers with damper constants *d*_1_, *d*_2_, respectively; *F*_*c*_ is the Coulomb frictions, and an exterior cos-wave force *F* is acting on object *m*_1_.

For modeling this mass-spring system, the exterior force *F* and the object position *x*_2_ are taken as the input-and-output signals *u* and *y*, respectively. Learning rate is 0.0001 in the Adam algorithm when offline training NN through 10,000 epochs. The NN is endowed with three inputs, including two presumed state variables *x* representing *x*_2_, ${\dot{x}}_{2}$ and system excitation *u*, and with one output representing the prediction $\hat{y}$ for *x*_2_.

The initial experimental values are: *m*_1_ = 20, *m*_2_ = 20, *k*_1_ = 1000, *k*_2_ = 2000, *d*_1_ = 1, *d*_2_ = 5, *F*_*c*_ = 0.05, sampling frequency *dt* = 0.05 s and the exterior force for generating training data is $F=4\cos (0.5\,\pi t)$. The training sample size is 2000 and takes up 158.34 s.

For testing the regulator, the ambient impacts are simulated through parameter changes in Table 4. The sampling frequency is the same, and the sample size is 3000. The normal Gaussian distribution has mean 0 and variance 10^−5^. The exterior force applied on the object is also changing, as listed in (42).

42 $$F=\left\{\begin{array}{ll}3\cos (0.5\pi t),\hfill\quad &[0,{t}_{1})\hfill\\ 1.5\cos (0.5\pi t),\hfill\quad &[{t}_{1},{t}_{2})\\ 1.0,\hfill\quad &[{t}_{2},{t}_{3})\\ -1.0,\hfill\quad &[{t}_{3},{t}_{4})\\ 0.6\cos (0.5\pi t)+0.4\cos (\frac{1}{3}\pi t),\hfill\quad &[{t}_{4},{t}_{5})\\ 0.6\cos (0.1\pi t),\hfill\quad &[{t}_{5},{t}_{6})\\ 2\cos (0.5\pi t),\hfill\quad &[{t}_{6},t)\end{array}\right.$$

**Table 4:** Mass-spring-damper system parameters varying in time

Data	System parameters
[0, *t*_1_)	original parameters, Gaussian noise in process and measurement
[*t*_1_, *t*_2_)	*m*_1_ = 0.98*m*_1_, *m*_2_ = 0.98*m*_2,_ *k*_1_ = 0.96*k*_1_, *k*_2_ = 0.98*k*_2,_ *d*_1_ = 0.96*d*_1_, *d*_2_ = 0.98*d*_2_, Gaussian noise
[*t*_2_, *t*_3_)	same parameters as in [*t*_1_, *t*_2_), Gaussian noise
[*t*_3_, *t*_4_)	*m*_1_ = 0.95*m*_1_, *m*_2_ = 0.98*m*_2,_ *k*_1_ = 0.96*k*_1_, *k*_2_ = 0.96*k*_2,_ *d*_1_ = 0.99*d*_1_, *d*_2_ = 0.99*d*_2_, Gaussian noise
[*t*_4_, *t*_5_)	same parameters as in [*t*_3_, *t*_4_), Gaussian noise
[*t*_5_, *t*_6_)	*m*_1_ = 0.98*m*_1_, *m*_2_ = 0.97*m*_2,_ *k*_1_ = 0.98*k*_1_, *k*_2_ = 0.96*k*_2,_ *d*_1_ = 1.99*d*_1_, *d*_2_ = 1.98*d*_2_, Gaussian noise
[*t*_6_, *t*)	${m}_{1}=\frac{{m}_{1}}{\sqrt{{m}_{1}}}$, ${m}_{2}={m}_{2}\sqrt{{m}_{2}}$, ${k}_{1}={k}_{1}-\sqrt{{k}_{1}}$, ${k}_{2}={k}_{2}-{k}_{2}^{2}$, *d*_1_ = 1.99*d*_1_, *d*_2_ = 1.98*d*_2_, Gaussian noise

*t*_1_ = 20 s, *t*_2_ = 40 s, *t*_3_ = 55 s, *t*_4_ = 70 s, *t*_5_ = 100 s, *t*_6_ = 125 s and *t* = 150 s.

**Fig. 10:**
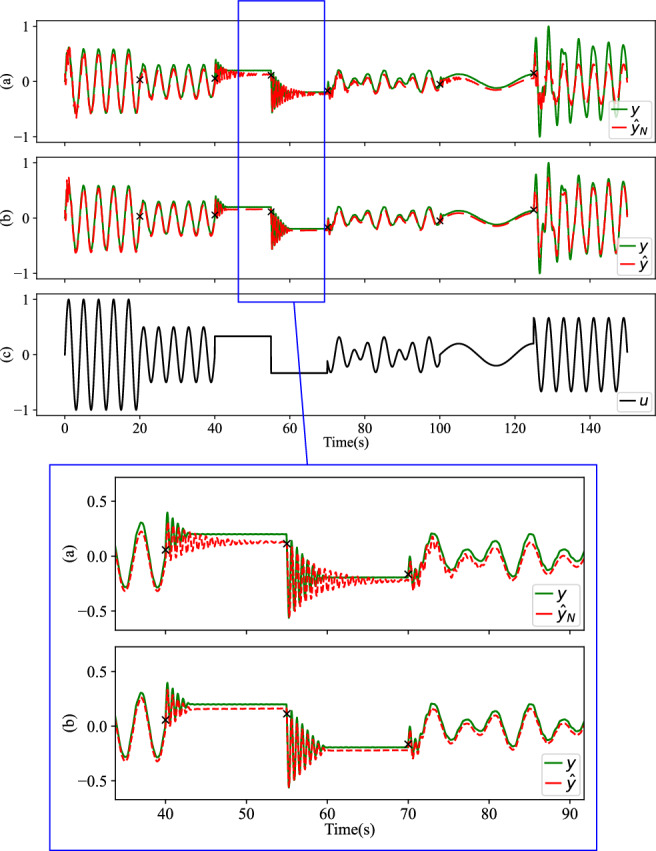
Mass-spring-damper system model adaptation. *y*, ${\hat{y}}_{N}$, and $\hat{y}$ represent the actual measurements, the pure pre-trained NN prediction and the regulated prediction, respectively. Physical parameter changes are marked with the ×. From top to bottom: **a** Using only the NN model, *R*^2^ = 0.794. **b** Using our recursive regulator, *R*^2^ = 0.965. **c** Input values to the model from (42). Data *u* and *y* are normalized in range [0, 1].

As illustrated in Fig. 10, the test results indicate that upon system impairment, the static-trained model is inadequate. Its accuracy diminishes further as the system parameters change and the input varies, leading to increasing errors in predictions (Fig. 10a, *R*^2^ = 0.794). By integrating the recursive regulator, predictions are substantially improved, restoring a high level of accuracy (Fig. 10b, *R*^2^ = 0.965) and extending the run time only modestly from 0.33 s to 3.45 s.

The cases studied above collectively demonstrate the efficacy of the proposed recursive regulator in adapting nonlinear models to system physically impaired conditions caused by sudden ambient impacts, all while maintaining high-speed performance.

## Conclusion

In this paper, we introduced an online model adaptation strategy named the recursive regulator, capable of modifying nonlinear models in real time for the systems encountering sudden ambient impacts. This regulator is designed to be recursive and rapid, making it well-suited for deployments where adaptive and cost-effective modeling is required.

The proposed regulator modifies the Koopman operator to permanently adapt the prediction trajectory upon encountering ambient impacts. It offers a solution capable of handling complex impaired models with remarkable accuracy, achieving up to *R*^2^ = 99%. This design offers an easy implementation, where the lightweight linear regulator can be implemented directly in the state space and is non-disruptive to the nonlinear predictor.

The versatility of this approach is demonstrated through its successful application to complex systems such as the electro-mechanical positioning system, the nonlinear RLC model, and the spring-mass-damper system. The test results across these systems underscore the method’s applicability and effectiveness in nonlinear model recalibration.

One of the notable advantages of the proposed method is its speed. It makes our method well-suited for on-device embedded systems that demand real-time rapid responses, ensuring operational stability and safety.

Looking ahead, upon restoring a predefined accuracy, the updating of the regulator can be disengaged to save processing energy. Experiments on more complex systems, i.e., higher system order, and more intensive ambient impacts can test the limit of this technique.

## Supplementary information

Supplementary Information

## Data Availability

The datasets generated and analyzed during the current study are available in the *github* repository, https://github.com/Jinming3/RecursiveRegulator.

## Code availability

All code used in this study is available at https://github.com/Jinming3/RecursiveRegulator.
