# Supplementary material for: Recursive regulator: a deep-learning and real-time model adaptation strategy for nonlinear systems
Source: Commun Eng. 2025 Aug 1;4:140. doi: 10.1038/s44172-025-00477-4 (PMC12316969; doi:10.1038/s44172-025-00477-4)
Supplement: Supplementary file 1 — Supplementary Information [file 44172_2025_477_MOESM1_ESM.pdf]

Recursive Regulator: A Deep-Learning and Real-Time Model Adaptation Strategy for Nonlinear Systems

Jinming Sun[1,*], Yanqiu Huang[2], Wanli Yu[1], and Alberto Garcia-Ortiz[1]

[1]Institute of Electrodynamics and Microelectronics, University of Bremen, 28359 Bremen, Germany.
[2]Faculty of Electrical Engineering, Mathematics and Computer Science, University of Twente, 7522 NH Enschede, The Netherlands.
[*]Corresponding author: jinming@uni-bremen.de

Supplementary note: functionality validation for recursive regulator

In this supplementary note, we evaluate the functionality of the proposed recursive regulator using the symbolic example presented in system (19) of the main manuscript. For clarity, the system is restated here:

$$x_t = \begin{bmatrix} x_{1,t} \\ x_{2,t} \end{bmatrix} \tag{1a}$$

$$\begin{bmatrix} x_{1,t+1} \\ x_{2,t+1} \end{bmatrix} = \begin{bmatrix} \gamma(x_{1,t} - x_{2,t}^2) + \beta u_t \\ \mu x_{2,t} \end{bmatrix} \tag{1b}$$

A typical way to validate a data-driven modeling approach is to compare the symbolic expressions of a known system to the ones produced by the method. This would involve:

- Identifying the system using a neural network (NN), without access to the exact Koopman lifting or true observables.
- Using the regulator to estimate model mismatches ($\bar{G}$ and ΔB) and comparing them to symbolic expressions.

Using such an academic example in a data-driven setting is challenging.

This system is indeed widely used to symbolically demonstrate the Koopman framework, owing to its finite-dimensional and exact Koopman lifting. However, in practice, if we simulate this system with a neural network-based lifting (without knowledge of the exact observables), several challenges arise.

1) Dimension mismatch and approximation limitations: A neural network structure with the common nonlinear activation (e.g., *ReLU*) cannot effectively represent polynomial features such as x_2^2 in a compact 3-dimensional space. Therefore, to approximate even this simple system, the NN must lift the state into a higher-dimensional space. As a result, the model mismatch terms $\bar{G}$ and ΔB do not align with the symbolic terms derived from the known analytical form.

2) Stability and data-driven limitations: To maintain stability, the system requires $|\mu| < 1$, causing state x_2 to decay exponentially over time. As a result, the dynamic behavior of x_2 effectively disappears after a short time. When this happens, the data-driven regulator, which relies on estimating Δx_2 from $\mu_0 x_2$, is left with very few signals to learn from. In essence, the contribution of x_2 becomes negligible, making it difficult—if not impossible—for the regulator to learn and infer meaningful updates.

To counteract this, one would need to introduce external excitation in x_2, which then destroys the system's Koopman finite dimension and requires lifting into a higher-dimensional space for linear approximation—again breaking the alignment with the symbolic structure.

3) Why our regulator does not require explicit identification of $\bar{G}$ and ΔB: While our design is to calibrate the Koopman operator with matrix $\bar{G}, \Delta B$ in the higher-dimensional space, a key contribution of our work is that our regulator operates directly in the original state space, without disrupting the originally trained Koopman predictor. This design is demonstrated in Fig. 2 of the manuscript. Instead of explicitly identifying $\bar{G}$ and ΔB, this regulator uses a data-driven linear SSM embedded PEM to perform the functionality of these update matrices. Specifically, it models the linear structure $G = \begin{bmatrix} C\bar{G}K^{-1} & C\Delta B \end{bmatrix}$. We choose this strategy because SSM handles multi-input systems naturally, and thus can construct the calibration $\bar{x}$ with the higher-dimensional data q^+.

As a demonstration of the regulator's effectiveness, we provide an alternative experimental setup to illustrate our approach, which is shown below.

NN modeling performance: We show that our NN-based lifting structure can model this system in its original condition.

To slow the decay of x_2, we set $\mu_0 = 0.99$ to near marginal stability, and $\gamma_0 = 0.5, \beta_0 = 5$. Input u is white noise with sampling frequency $0.5\,\mu s$ and bandwidth $60\,kHz$. As illustrated below in Fig. 1, the size of 500 samples are collected before the dynamics vanish, 300 samples are used for training and 200 for testing. The training is conducted through 10000 epochs with a learning rate 0.0001. Despite the limited data, the NN model achieves an accuracy of $R^2 = 0.946$.

Testing the regulator with known basis: Assuming that the three true lifting basis are known, we integrate

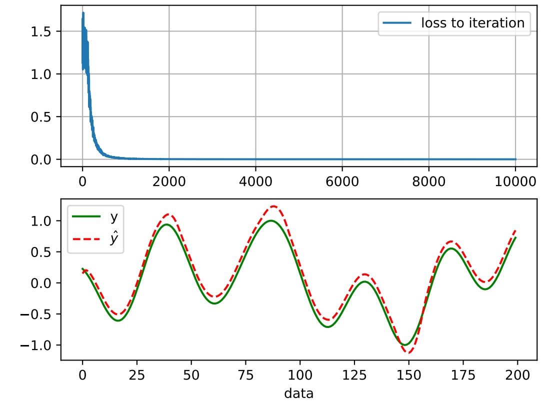

Figure 1: NN structure models the nonlinear system in original condition.

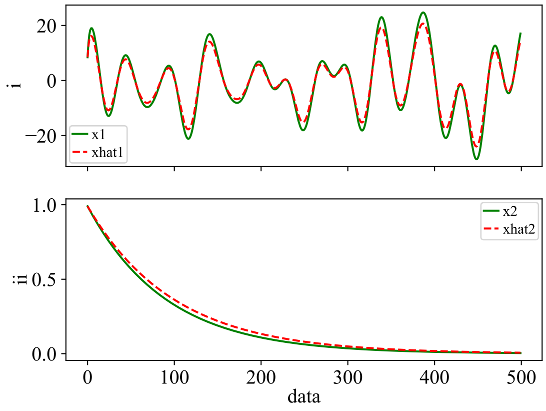

Figure 2: Parameter variation causes prediction discrepancy.

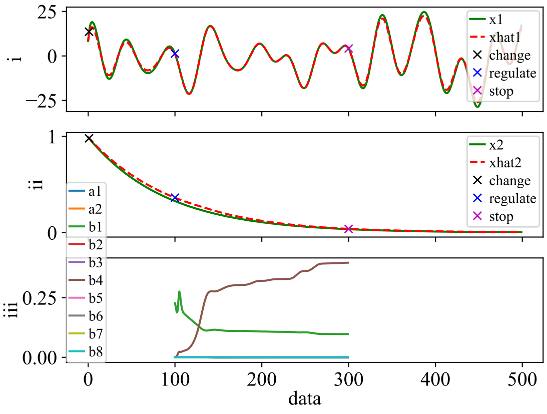

Figure 3: The regulator is integrated between time 100 and 300. The model accuracy is improved and the elements of the regulator reach stable values.

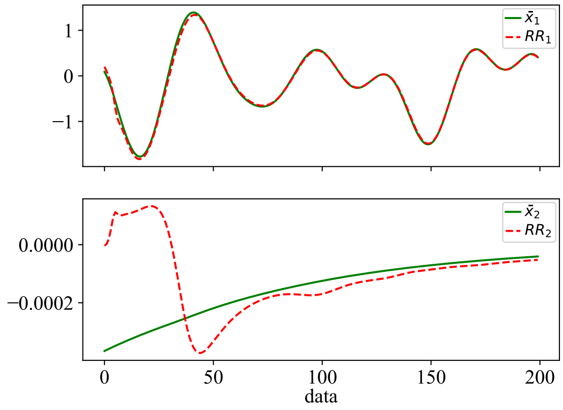

Figure 4: The actual model mismatch $\bar{x}$ and recursive regulator (RR) compensation.

the recursive regulator to assess model updating under parameter variations.

Parameter changes occur at $t = 1$ with $\Delta\mu = -0.001, \Delta\gamma = 0.05, \Delta\beta = 0.4$. The regulator is integrated from $t = 100$ and stops updating at $t = 300$.

The system setup is not ideal. The parameter variations are intentionally large for demonstration purposes. Since the system state decays fast, the number of meaningful samples is limited, which poses a challenge for the regulator to converge properly.

As depicted in Fig. 2, the original model prediction $xhat$ exhibits discrepancies once the parameters change. In Fig. 3, between the time 100 and 300, the regulator significantly improves the model prediction with only a few data. When the regulator stops updating, the accuracy slightly decreases but remains better than the pre-regulation state. The internal elements of $\mathbb{B}$ in the regulator are reaching stable values after the parameter variations (Fig. 3-iii).

Visualizing model mismatch and regulator compensation: We visualize the model mismatch $\bar{x} = G[q^+, u]^T$ and the compensation achieved by the regulator. As shown in Fig. 4, with only 200 data available, the regulator compensates for the model mismatch.

When assuming $\mathbb{A}$ is singular, we can get $\mathbb{B} \approx G$.

The regulator identification is

$$\mathbb{B} = \begin{bmatrix} 0.0975 & 0 & 0 & 0.3969 \\ 0 & -0.0013 & 0 & 0 \end{bmatrix} \tag{2a}$$

$$\mathbb{A} = \begin{bmatrix} 0 & 1 \\ 0 & 0 \end{bmatrix} \tag{2b}$$

The analytic of G is

$$G = \left[\begin{array}{ccc|c} \frac{\Delta\gamma}{\gamma_0} & 0 & 0 & \Delta\beta \\ 0 & \frac{\Delta\mu}{\mu_0} & 0 & 0 \end{array} \right] = \begin{bmatrix} 0.1 & 0 & 0 & 0.4 \\ 0 & -0.001 & 0 & 0 \end{bmatrix} \tag{3}$$

which illustrates and validates the functionality of our approach.
